# Phytoremediation of Potentially Toxic Elements from Contaminated Saline Soils Using *Salvadora persica* L.: Seasonal Evaluation

**DOI:** 10.3390/plants12030598

**Published:** 2023-01-29

**Authors:** Amtul Mujeeb, Zainul Abideen, Irfan Aziz, Nadia Sharif, Muhammad Iftikhar Hussain, Asad Sarwar Qureshi, Hsi-Hsien Yang

**Affiliations:** 1 Muhammad Ajmal Khan Institute of Sustainable Halophyte Utilization, University of Karachi, Karachi 75270, Pakistan; 2 Department of Biotechnology, Woman University Mardan, Mardan 23200, Pakistan; 3 Department of Plant Biology & Soil Science, Campus Lagoas Marcosende, Universidad de Vigo, 36310 Vigo, Spain; 4 Sustainable Natural Resources Management Section, International Center for Biosaline Agriculture, Dubai 14660, United Arab Emirates; 5 Department of Environmental Engineering and Management, Chaoyang University of Technology, Taichung 413310, Taiwan

**Keywords:** *Salvodora persica*, salinity, acclimation, chemical toxicity, littoral, phytoextraction

## Abstract

Plants in coastal ecosystems are primarily known as natural sinks of trace metals and their importance for phytoremediation is well established. *Salvadora persica* L., a medicinally important woody crop of marginal coasts, was evaluated for the accumulation of metal pollutants (viz. Fe, Mn, Cu, Pb, Zn, and Cr) from three coastal areas of Karachi on a seasonal basis. Korangi creek, being the most polluted site, had higher heavy metals (HM’s) in soil (Fe up to 17,389, Mn: 268, Zn: 105, Cu: 23, Pb: 64.7 and Cr up to 35.9 mg kg^−1^) and *S. persica* accumulated most of the metals with >1 TF (translocation factor), yet none of them exceeded standard permissible ranges except for Pb (up to 3.1 in roots and 3.37 mg kg^−1^ in leaves with TF = 11.7). Seasonal data suggested that higher salinity in Clifton and Korangi creeks during pre- and post-monsoon summers resulted in lower leaf water (*ΨW*_o_) and osmotic potential at full turgor (*ΨS*_o_) and bulk elasticity (*ε*), higher leaf Na^+^ and Pb but lower extractable concentrations of other toxic metals (Cr, Cu, and Zn) in *S. persica*. Variation in metal accumulation may be linked to metal speciation via specific transporters and leaf water relation dynamics. Our results suggested that *S. persica* could be grown on Zn, Cr and Cu polluted soils but not on Pb affected soils as its leaves accumulated higher concentrations than the proposed limits.

## 1. Introduction

Trace metal pollution is known to alter the quality of air, water, and soil, making the environment undesirable as it is a major threat to ecosystems and human society [1,2]. In the modern era, the assessment of potential chemical toxins released from wastewater has gained attention [3]. Research on hazards caused by trace metal contamination and solid waste management is essential for systemic health effects [2]. Several methods have been suggested to prevent the entry of heavy metals into the food chain, including the use of chemicals, e.g., surfactants, rhamnolipid solutions, etc., [4], despite their detrimental effect on the environment. Moreover, soil salinity has also increased the problem of food production in agricultural settings [5]. In view of vegetation destruction, contamination in terrestrial and aquatic ecosystems, and the ever-increasing human population, food security has become a major challenge for sustainable development goals (SDGs) [6]. The release of toxic chemicals in the natural environment by anthropogenic activities could supplement their entry into edible plant products beyond recommended limits which is a major concern for human health disorders [7]. Moreover, the enrichment of soils with heavy metals also brings about changes in soil physio-chemical characteristics, which may decrease the growth and productivity of economically important crops [8]. For cleaning soils that are contaminated with trace metals, various methods are being practiced, e.g., soil solidification, chemical leaching, soil oxidation, and reduction, etc., yet these methods are expensive and detrimental to the environment [3]. Phytoremediation is a sustainable approach in the sense that it does not require chemicals for cleaning hence rendered environmentally safe [6]. Therefore, phytoremediation as a cost-effective green technology is suggested.

Phytoremediation of salts using halophytes is gaining special attention for its environment-friendly method [9]. However, recent studies suggested that halophytes growing in coastal areas may also be used for phytoremediation of polluted lands as some of them use similar physiological strategies for removing metals [8,10]. Salt-resistant halophytes of the coasts could play an important role in metal absorption and translocation, thereby helping clean metal-polluted soils [11]. Studies suggested that halophytes can resist heavy metals, for example, Cd, Pb, Zn, Cu, etc. [8,12,13,14,15]. However, reports on cross-tolerance mechanisms of heavy metals with salinity are scant. Ecophysiological studies on salt and metal resistance of *Atriplex atacamensis* [16], *Suaeda maritima* [17], *Tamarix gallica* [18], etc., indicated the phytoremediation ability of halophytes. Moreover, halophytes for important bioactive metabolites with commercial value may be cultivated for food, fodder, forage, fuel, and medicinal crops on saline lands [6,7,8,9,10,11,12,13,14,15,16,17]. Hence, halophytes can be used as alternative crops and may be potential candidates for phytoremediation [19]. However, careful selection of species is needed as some of them may accumulate higher concentrations of trace metals in their edible parts, more specifically in leaves [20]. Plants with normal permissible limits of metals in leaves and other edible parts, such as seed/fruit, etc., may be used as food, fodder, etc., but those with toxic levels of metals are not advisable to be used [21]. Several halophytes belonging to different taxa have the potential to remediate soils polluted with trace metals as well [21]. Recent research articles mainly focused on perennial plants with higher above-ground biomass that may be harvested from time to time [10,22]. However, tree crops may also provide great opportunities for their economic potential besides serving the purpose of phytoremediation.

*Salvadora persica* L. is a facultative halophyte that normally grows in semiarid regions of the Middle East [23] and can survive in highly saline lands along coastal regions [24]. This species has the ability to grow and adapt to different habitat types (dunes, Sabkha, and marshes) and can resist frequent tidal inundations near the coasts [24]. *Salvadora persica* could grow well in up to 25 dS m^−1^ (about 250 mM NaCl) under natural conditions [24] and is classified as a salt-accumulating species [25]. In Pakistan, *S. persica* L., grows all along the Indus plain and sandy desert tracts of coastal and near coastal areas of Sindh and Baluchistan [26]. This plant has traditionally been used as medicine for various ailments like asthma, cough, rheumatism, scurvy, etc. [27]. Its tender leaves are used as salad and pickles in Pakistan [25]. The fruit is also edible with a sweet taste, considered carminative and diuretic, and has stomachic properties. Twigs of this plant are used as miswak for cleaning teeth [28].

**Figure 1:**
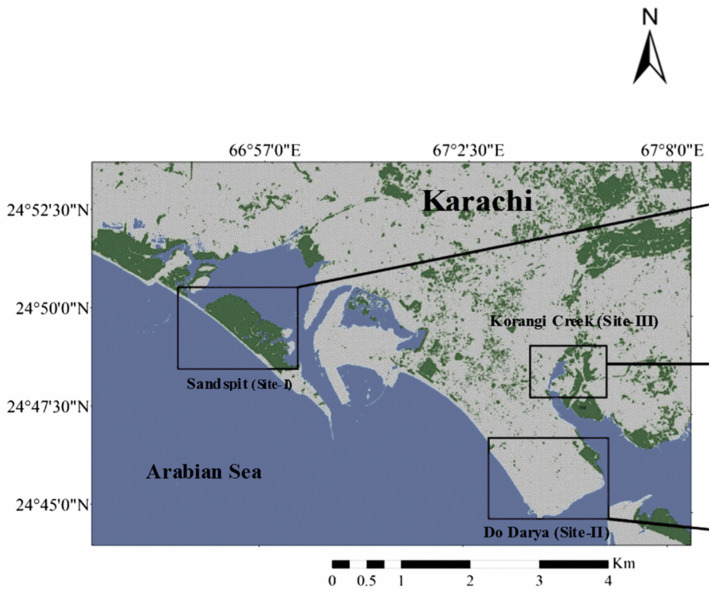
Map of the studied area with highlighted locations (Sandspit = site I; Clifton (near Do-Dariya = site II and Korangi = site III).

The potential of *S. persica* for the phytoremediation of salts is well established [28,29]. Its immense value as a potential seed oil crop is also well known [24,25,26,27,28,29,30]. Roots of *S. persica* are typically known to remove wastewater metals (Cd, Pb, Zn, Cu, Ni, As, etc.) due to their high adsorption capacity [31,32]. However, there are few reports on both salt and metal tolerance of this tree as a non-conventional crop [33]. Studies on the metal tolerance of *S. persica* under field conditions and their bio-concentration (BCF) and translocation (TF) factors are scant. Therefore, understanding the fate of trace metals in plant tissues is of crucial importance for ascertaining phytoremediation. This study aimed to evaluate the phytoremediation potential of *S. persica* to six trace metals (Pb, Cr, Cu, Fe, Mn, and Zn) and its water relation dynamics under fluctuating salinity in field conditions. Three coastal areas (Sandspit, Clifton, and Korangi creek near the Industrial area) were selected (Figure 1). Seasonal fluctuations (winters, pre-monsoon summers, and post-monsoon summers) in metal concentration and BCF and TF in *S. persica* were recorded. The following hypotheses were tested: (1) Heavy metal accumulation in *S. persica* would be seasonal and site-specific (2) Spatio-temporal changes in soil salinity would alter the shoot metal accumulation in *S. persica*.

## 2. Results

### 2.1. Seasonal Changes in Soil Characteristics at Different Study Sites

**Figure 2:**
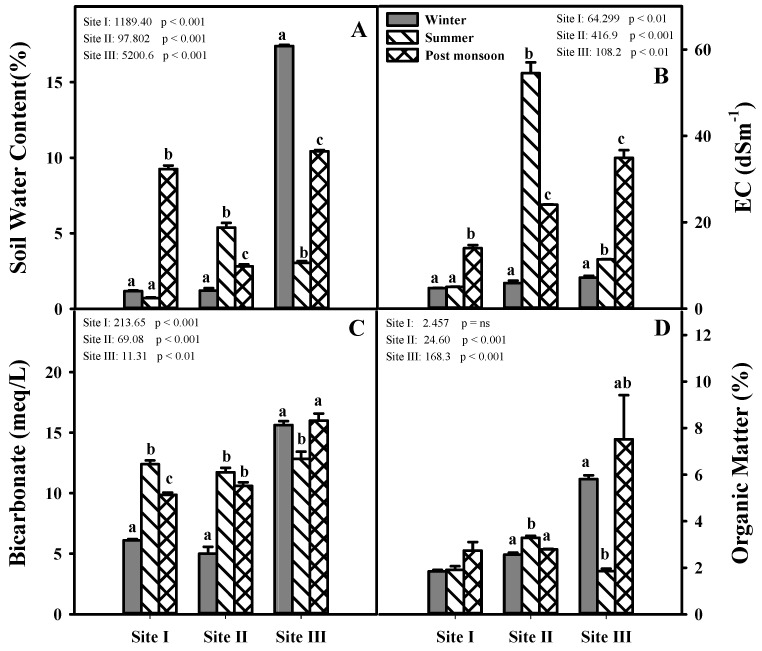
Water content (**A**), Electrical conductivity (**B**), Bicarbonates (**C**) and Organic matter (**D**) in soil of test species at Sandspit (Site I), Clifton (Site II) and Korangi (Site III). Different letters on error bars indicate significant differences among seasons on the same site (*n* = 3) at *p* ≤ 0.05.

Seasonal and spatial physiochemical characteristics in soils of *S. persica* are summarized in Figure 2. Results of ANOVA/Welch’s tests showed that % soil water, electrical conductivity (EC), and bicarbonates (HCO_3_) differed significantly (*p* < 0.001) in all study sites (Sandspit, Clifton and Korangi) among different seasons (Figure 2). Soil organic matter (OM) ranged between 2–9%, with the highest values (9%) at Korangi in post-monsoon summers (Figure 2). However, no change in soil OM was found at Sandspit (Figure 2). The soil EC at study sites notably increased with the increase in the frequency of tidal inundation during summers, reaching up to 56 dS m^−1^ in pre-monsoon at Clifton and 38 dS m^−1^ in post-monsoon at Korangi (Figure 2). There was, however, no change in soil pH with changing seasons (data not given) and it remained between 7.2 to 7.5 at Sandspit and Clifton and 7.9–8.4 at Korangi.

### 2.2. Seasonal and Spatial Variation in Soil Sediment Metal Ions

Extractable metals (mg kg^−1^ DW) in *S. persica* soils differed significantly (*p* < 0.05; Bonferroni/Games-Howell tests) at all study sites in different seasons (Table 1). Among six tested metals, Fe was the highest in soil sediments (reaching up to 17,390 mg kg^−1^ at Korangi in summers), and an overall metal availability was in the following order at sites Sandspit and Clifton: Fe > Mn > Zn > Pb > Cr > Cu (Table 1). Korangi, with the highest level of pollution, represented soil metals in the following order: Fe > Mn > Zn > Cr > Pb > Cu, with generally higher values in winter (Table 1). Values for both soil Na^+^ and K^+^ were generally higher at the same site (Korangi) in pre- and post-monsoon summers, but at Sandspit, Na^+^ was the highest in winter (Table 2).

### 2.3. Seasonal Flux in Extractable Metal Ions in Plant Tissues

Extractable metal concentrations in the roots and leaves of *S. persica* are presented in Table 1. The root metal concentrations in *S. persica* were significantly higher (*p* < 0.05; Bonferroni/Games-Howell tests) in pre- and post-monsoon summers (except for Fe at Sandspit and Cu at Clifton in winter). Root metals were in the following order at study sites: Fe > Zn > Mn > Cu > Pb > Cr (Table 1). Except for Cu (13.5 mg kg^−1^) and Pb (8.2 mg kg^−1^) at Sandspit (post-monsoon) and Zn (winter) at Sandspit (50.6 mg kg^−1^), most of the study sites had lower Zn in *S. persica* with <1 BCF values for metals (Cr, Mn, and Fe) (Figure S1; Supplementary Data). Root Na^+^ and K^+^ in *S. persica* were the highest in post-monsoon summers on all study sites (Table 2). However, BCF values were the highest at Sandspit (Figure S1; Supplementary Data). Metal concentrations in *S. persica* leaves also varied significantly (*p* < 0.05; Bonferroni/Games-Howell tests) among seasons as well as study sites (Table 1). Barring Cu and Cr, leaf metal concentrations were generally higher in pre- and post-monsoon summers and were in the following order: Fe > Mn > Zn > Cu > Pb > Cr (Table 1). Leaf Fe and Mn were significantly higher during pre-monsoon summer on all study sites, Zn at Clifton in pre-monsoon and at Korangi in post-monsoon, Cu in winter on all study sites, and Pb at Sandspit in pre-monsoon summers (Table 1). Leaf Na^+^ and K^+^ increased during summers with increased tidal inundations in the littoral zones of *S. persica* and the highest values were recorded in post-monsoon summers (Table 2). The translocation factor (TF) for most of the metals (except Cr at Clifton, Pb at Korangi, and Cu at Sandspit in summers) as well as Na^+^ were >1 on all study sites (Figure S1; Supplementary Data).

### 2.4. Seasonal Fluctuations in Leaf Water Relation Parameters at the Study Sites

Water relation parameters in *S. persica* leaves are presented in Table 3. Leaf succulence (mg g^−1^ DW) in *S. persica* was generally higher at Clifton, followed by Korangi and Sandspit, with the maximum values in pre-monsoon summers (Table 3). Pressure-volume (PV) curve analysis showed that at full turgor, leaf water (*ΨW*_o_), as well as osmotic potential (*ΨS*_o_), was higher in post-monsoon summers on site I (Table 3). However, both *ΨW*_o_ and *ΨS*_o_ were lowest (more negative) at Korangi, followed by Clifton in pre-monsoon summers. Similar patterns were noted for leaf osmotic potential at the turgor loss point (ΨTLP) as well as bulk elasticity (*ε*) representing leaf rigidity (Table 3).

## 3. Materials and Methods

### 3.1. Description of Study Sites

Karachi has a semi-arid climate with a low annual rainfall from July to September (average of <220 mm). Both winters and summers are mild, with average temperatures of ~20 °C (winters) and ~32 °C (summers) [34]. Karachi coast experiences mostly dry winters with low tidal inundations and high tides during pre- and post-monsoon summers. A sampling of soil and plants was done from three littoral zones of the Karachi coast (Figure 1), the descriptive data of which are mentioned below:

Sandspit: It is located on the northwest coast (24.81° N; 66.94° E) near (about 4 km) Mauripur S.I.T.E. (Sindh Industrial Trading Estate) that receives waste partially from functional sewage treatment via the Lyari river [35]. Waste from 3 fertilizers and some of the tannery factories is also dumped near “Shamspir” and “Kakapir” tributaries;Clifton (near Do-Dariya) is about 3–4 km away from the Korangi Industrial area (24.75° N; 67.08° E) that receives effluents from the Korangi Industrial area via the tributaries of the Malir river [36], and Korangi creek is nearest (about 1 km) to the Industrial area (24.78° N; 67.13° E) that receives discharges from tanneries via the Malir River [37]. Domestic sewage and pesticides/chemicals are also dumped from >500 factories via rain run-off [38,39].

### 3.2. Sample Collection

Three sample plots within a ~25 m radius and 50 m apart were marked in each area for soil and plant samples in three seasons. Plants and soil samples were pooled (2 of each from a plot) to make a replicate (*n* = 3), as mentioned in Mujeeb et al. [10] (2020). For physiological measurements (viz., Fe, Mn, Zn, Cu, Pb, and Cr metals, Na^+^ and K^+^, water relation parameters, etc.). Plants of almost equal size (3–4 m in height) were tagged in the field for the seasonal studies at each site. Data for winter were collected between the middle of January to February, the pre-monsoon summer in the middle of May to June, and the post-monsoon in the middle of September. Soil samples of the test species were carefully drawn from the root zones of *S. persica* with the help of a soil corer (up to 15 cm depth). Root samples of secondary branches were dug from the soil, and fully expanded leaf samples (three of each from the second node of the middle canopy of the branch) were directly separated from the trees. All samples were kept in vacuumed plastic bags, placed in iceboxes, and transported to the laboratory.

### 3.3. Plant Analyses

The leaf and root samples of *S. persica* were carefully separated, cleaned with distilled water in an ultrasonic bath (Elmasonic E 30 H, Singen, Elma Schmidbauer GmbH, Germany), and gently blotted to remove surface water. Fresh weights were taken immediately, followed by drying at 60 °C in a forced draft oven for 48 h or till the constant weights were achieved [10].

### 3.4. Soil Analyses

Samples were sieved with a 0.02 cm mesh, and fresh weights were immediately taken. Then, soil samples were oven dried at 105 °C for 48 h, and the percentage of soil water (SW) was calculated, as described in Mujeeb et al. [10]. Oven-dried soil samples were ashed in a Furnace at 550 °C for 3 h. The percentage (%) of soil organic matter (OM) was calculated by the method described in Mujeeb et al. [10]. Oven-dried soil samples were kept in a Furnace at 550 °C for 3 h for ashing. Soil OM (%) was calculated by subtracting soil ash weight from oven-dried weight of the soil using the following formula:

OM = Oven-dried weight of soil sample − Ash weight of soil sample × 100/Oven-dried weight of soil sample

Ten grams of oven-dried soil was mixed in 50 mL of distilled water to make a Soil—Water mixture (1:5), shaken on a soil shaking machine for 1 h, kept aside for 24 h, and finally filtered with the help of a Whatman filter paper (pore size 8 µM). The soil electrical conductivity (EC) and pH were determined on filtrates of soil samples with the help of a pH/EC meter (CPC-401, Elmetron, Zabrze, Poland). The method of Richards [40] was used for the determination of soil Bicarbonates (HCO_3_).

### 3.5. Heavy Metals (Fe, Mn, Pb, Cu, Zn, and Cr) and Ion (Na^+^ and K^+^) Analyses

Soil sediments and plant samples (roots and leaves) were digested according to Otte et al. [41] with minor modifications [10]. Dried samples were finely powdered in a ball mill (MM 400, Retsch, GmbH, Germany) prior to acid digestion. All samples were digested by the method of Mujeeb et al. [10]. The plant and soil sample extracts (4–5 mL) were filtered using a Whatman No. 42 filter paper (8 µM pore size) and diluted with distilled water to a total volume of 20 mL. Trace metals (described above) were determined by the flame Atomic Absorption Spectrometry (Hitachi Z-8000, model 1984, Tokyo, Japan), with the help of different lamps (Fe, Mn, Zn, Cu, Cr, and Pb) and Na^+^ and K^+^ by Digital Clinical Flame photometer (model I-65, Intech Technologies, Singapore). Quality assurance for precision and accuracy and percentage metal recoveries and LOD (Limit of Detection) was maintained as in Mujeeb et al. [10].

### 3.6. Bio-Concentration Factor (BCF) and Translocation Factor (TF)

Bio-concentration factor (BCF) and translocation factor (TF) were calculated [42] using the formulas:

BCF = ECM/ECS(1)

TF = EML/ECM(2)

where, ECM is the extractable concentration of metals (mg kg^−1^) in roots; ECS is the extractable metal concentration in the soil; and EML is the extractable metal concentration in leaves.

### 3.7. Water Relation (PV-Curve Derived) Parameters

Fully expanded young leaves (from the second node) of the middle branches of *S. persica* were selected for PVC measurements. Leaf branches were hydrated overnight by covering them with plastic bags. Following complete hydration, bags were removed in the early morning (pre-dawn), leaves were cut from the branches, and their hydrated weights were taken immediately. A 5 mm leaf disc was then punched from each sample and quickly inserted in a sample chamber of a Wescor HR-33T thermocouple psychrometer (Wescor, Inc., Logan, UT, USA) to minimize air exposure [43]. Water potential was determined after thermal equilibration. Between 8–10 repetitive measurements, leaf samples (along with leaf discs) were subjected to bench drying (1–20 min) and re-inserted in the sample chamber to note subsequent water potential readings. A Pressure-volume curve (PVC) was obtained by the method of Shoukat et al. [43], and the following water relation parameters were derived: water and osmotic potential at full turgor (*ΨS*_o_ and *ΨW*_o_), water potential at turgor loss point (*ΨTLP*), and bulk elastic modulus (*ε*) (Bartlett et al., 2012) [44].

### 3.8. Statistical Analyses

Statistical analysis was performed using SPSS version 16 for windows. Mean ± SE values were calculated; data were checked for variance homogeneity prior to the ANOVA and post-hoc (Bonferroni) tests. The data that represented unequal variance were subjected to the Welch statistics and Games-Howell post-hoc test to determine significant differences among mean values.

## 4. Discussion

Plants of the littoral zones can survive under salt and metal stress [8,45], and they bear different mechanisms to cope with seasonal fluctuations in the environment [10]. In this study, we tried to elucidate the phytoextraction ability of trace metals in *Salvadora persica* under fluctuating soil salinity and metal composition in different seasons. Some of the soil physiochemical parameters, e.g., organic matter (OM), bicarbonates (HCO_3_), and extractable metal concentrations (Mn, Zn, Cu, Pb, etc.), suggested that Korangi creek was the most polluted site, followed by Clifton and Sandspit, as has been reported in earlier findings [10]. The major cause of the increased level of pollution at Korangi creek is its closest vicinity (about 1 km) to the industries, which outpours waste material into the Malir river and carries it to coastal zones via several streams [10]. An average of ~350 MGD (million gallons per day) outflow from the Malir river directly into the sea has been reported in earlier studies [10,36]. On the other hand, Sandspit beach is about 4 to 5 km away from the S.I.T.E (Industrial area), mostly affected by sewage dumping and some of the tanneries near Mauripur [8]. Although soil salinity in terms of EC and Na^+^ increased at Clifton and Korangi in post-monsoon summers, generally lower values of metals in *S. persica* soil (except Zn and Pb at Korangi) may either be related to plant uptake or dilution effect owing to the tidal influx/rain runoff [10,46,47]. In the first instance, salinity (EC) could result in metal chloro-complexation, which generally causes enhanced biosorption of metals by plant roots [48]. Moreover, the soil pH did not deter metal adsorption as it remained similar (alkaline) throughout the study period (7.2–8.4), as reported in the literature [10,32]. It is generally believed that some of the root exudates may also cause changes in soil metal bioavailability [6]. However, metal transfer from root to shoot system requires specific transporters [14] along with water relation dynamics of the plant [8]. Nevertheless, it is important to identify strategies employed by plants for metal biosorption and their phytoextraction potential, as well as for efficient decontamination of polluted soils [6,49]. However, the use of halophyte plants with higher metal concentrations is not advisable for edible purposes [21]. Nevertheless, if any plant could grow and phytostabilize such metals while restricting shoot metal translocation below toxic levels, it may be recommended for edible purposes with added benefits of soil remediation [50,51].

Among the tested heavy metals in *S. persica*, root Fe was the highest in winter (919 mg kg^−1^), which exceeded the normal plant limit (450 mg kg^−1^) [50] at Sandspit, where tannery waste is disposed of [10]. However, at Clifton and Korangi, higher root Fe was noted in summer, which may be related to excessive outflow under the tidal influence or formation of root iron plaques. A higher influx of metals into the root system is known to be associated with specific Fe metal transporters [51]. However, TF > 1 for Fe at Sandspit and Clifton also showed subsequent translocation to leaves. Iron (Fe) concentrations in leaves were found under acceptable limits and never crossed beyond toxic (>600 mg kg^−1^) levels in any season on all study sites. Root Mn was also the highest in pre-monsoon summers with <1 BCF on all study sites but never exceeded the permissible limit of 50 mg kg^−1^ in any season [49,50,51]. Both Fe and Mn are micronutrients and are essentially needed by components of plant antioxidant enzymes in several physiological and biochemical functions [52,53,54]. If found well within the suggested ‘normal ranges’, Fe and Mn could be beneficial for plant growth, otherwise damaging in the case of toxic concentrations [54]. Similarly, Zn is also a micronutrient that could be helpful in chlorophyll synthesis and plant growth within standard permissible ranges [50]. The adequate level of Zn is <20 mg kg^−1^ [55], while the maximum limit is up to 60 mg kg^−1^ [50]. In this study, Zn in both the root and leaf remained <60 in *S. persica* in all seasons on all study sites, although leaf Zn markedly decreased in summer with increases in soil salinity.

Among other tested metals, Cu and Cr serve as co-factors for certain enzymes [56,57] and are involved in many redox reactions [10]. However, at the functional level, the toxicity of Cu in plants (with >40 mg kg^−1^) is still unclear [10], except that it may lead to stunted growth in many crops [58]. Lead (Pb), on the other hand, is thought to be a non-essential element for plant growth [59]. Therefore, the standard Pb range is 0.3 to 0.6 mg kg^−1^ of dry weight, and values higher than this range could prove toxic for many plants [60]. In this study, *S. persica* consistently accumulated Cu well within acceptable ranges but with preferably higher extractable concentrations in roots than leaves. Furthermore, the transport of Cu to leaves also decreased with the increases in soil salinity during summer (pre- and post-monsoon). The accumulation of Pb in both roots and leaves was consistently higher than their normal limits as described above. The higher Pb in *S. persica* roots during post-monsoon summers may be attributed to Pb binding at the root surface during high tides [61,62,63]. However, the higher leaf Pb in winter at Sandspit and Clifton in pre-monsoon summers could be a result of the symplastic transport to shoot, possibly due to the immobilization of Pb by root cell wall components [64]. Salt-accumulating species are known to have a higher leaf Pb, and *S. persica* appears no different from other coastal halophytes [65,66,67]. Chromium (Cr) is also a non-essential metal, serving as a co-factor for a few enzymes [68] if found in concentrations <5 mg kg^−1^ [50]. Negi [69] reported Cr in leaves up to 23 mg kg^−1^ in *S. persica*; however, in this study, the highest leaf Cr was 3.37 at Sandspit in winter. Despite the same report, the leaf Pb was up to 54 mg kg^−1^ in *S. persica*; in our case, it ranged between 1.1–4.66 mg kg^−1^ during different seasons. Apparently, *S. persica* leaves accumulated only Pb among tested metals with higher values than the suggested (0.6 mg kg^−1^) limit.

Leaf Na^+^ content increased during pre- and post-monsoon summers in *S. persica* at Clifton and Korangi creeks along with the Pb. Higher Na^+^ content in leaves with exceptionally higher TF (5–10) indicated that *S. persica* is a Na^+^ hyperaccumulator, though increased leaf succulence may help in resisting salt stress [70,71,72]. Moreover, the higher leaf K^+^ in both summer seasons and maintained leaf Na/K (~4–7) ratio in *S. persica* suggested efficient osmotic adjustments [24,29]. It is also reported that the increased leaf succulence further enhances Na^+^ sequestration in the vacuolar compartment carrying along with it certain metals [73,74,75]. Salt secretors, on the contrary, efficiently remove salts along with Pb in the apoplast which may be excreted out through specialized glands [76]. In this aspect, salt accumulators seem to adapt similar physiological and biochemical traits for salt and metal stress. However, vacuolar sequestration of Pb in *S. persica* requires experimental evidence. Low leaf water potential (more negative) at full turgor (*ΨW*_o_), as well as at turgor loss point (*ΨTLP*) and bulk elastic modulus in samples collected from Clifton and Korangi during winters, pointed towards a decreased cell hydraulic capacitance. Hydraulic capacitance seemed to increase with the onset of summers on both sites with the increase in soil salinity [72]. Changes in water relations are known to trigger the regulation of aquaporin gene expression, which may indirectly contribute to reductions in water loss [77]. Furthermore, the deposition of metals in cell walls could result in their thickening (increased bulk elasticity), which may cause reduced rates of water movement via the vacuolar continuum [77]. Marginal increases in bulk elasticity during winters in *S. persica* indicated deposition of some metals, e.g., Cr, Cu, and Zn, which were lower in leaves during summers. These findings suggested that seasonal and spatial increases in soil salinity could have promoted decreasing metal transfer to *S. persica* foliage. Increases in soil salinity are also associated with the increase in some of the proteinogenic amino acids (e.g., Glutamine, Aspargine, etc.), and these are potentially known to be metal chelators [28]. Although it is not confirmed, the decreased metal transfer may also be associated with such kinds of active chelators among other metal transporters. It may be summed up that seasonal flux in extractable trace metal may be related to water relation dynamics besides metal speciation associated with specific transporters. However, further elaborative experiments are needed with an emphasis on dose-dependent salt and metal stress in halophyte trees such as *S. persica*. In view of trace metal selectivity, lab trials to check the extent of metal tolerance should be designed for more realistic and applicable measures. Moreover, since the fruit of *S. persica* is also edible, it would be interesting to know the extent of trace metal accumulation, which has not been reported in prior studies. This study has also provoked us to ask a few more questions: (1) How does the salinity affect the transport of different metals to shoots via root, especially in tree halophytes? (2) What anatomical differences are caused at root and leaf levels for metal transport under saline conditions? (3) Is there any proteinogenic amino acid involved that promotes metal chelation, thereby affecting metal transfer? Information on metal-binding proteins/transporters at the root and leaf level would help improve bioremediation processes to get rid of toxic metals from the soils.

## 5. Conclusions

This study revealed that *S. persica* could remove multiple trace elements along with salts. *Salvadora persica* may be effectively grown on soils polluted by Zn, Cr, and Cu. Since most of the metals are phytostabilized and do not exceed toxic levels, leaves may be used for edible purposes, except for Pb, which was higher than the normal suggested limits. Therefore, on Pb-polluted soils, the plant may be solely used for phytoremediation while avoiding it for edible purposes. Based on the metal specificity of the species, extensive lab studies with molecular insights would further enhance bio-monitoring programs for saline and polluted wastelands in a sustainable way.

## Figures and Tables

**Table 1:** Extractable metal concentration (mg kg^−1^) in soil, root, and shoot of *Salvadora persica* at Sandspit (Site I), Clifton (Site II) and Korangi (Site III). Different letters on error bars indicate significant differences among seasons on the same site (*n* = 3) at *p* ≤ 0.05 (Bonferroni/Games-Howell tests wherever applicable). Asterisk (*) in mean values represent the most significant data.

		Soil			Root			Leaves		
Sites	Metal	Winter	Summer	Post Monsoon	Winter	Summer	Post Monsoon	Winter	Summer	Post Monsoon
Site I	Cr	19.6 ± 0.6 a	9.93 ± 0.2 b	3.70 ± 0.1 c	1.25 ± 0.03 a	3.09 * ± 0.0 b	1.25 ± 0.1 a	3.37 * ± 1.8 a	0.01 ± 0.0 c	0.65 ± 0.1 b
Site II		23.2 ± 1.5 a	9.88 ± 0.2 b	5.84 ± 0.2 b	1.25 ± 0.0 a	2.04 * ± 0.03 b	0.90 ± 0.2 a	0.50 * ± 0.1 a	0.45 * ± 0.1 a	0.01 ± 0.0 b
Site III		35.9 * ± 0.3 a	8.8 ± 0.04 b	19.9 ± 0.3 c	2.64 ± 0.1 a	1.09 ± 0.2 b	3.03 * ± 0.02 a	2.22 * ± 0.4 b	2.15 ± 0.2 b	1.35 ± 0.3 a
Site I	Pb	4.27 ± 0.3 a	12.3 ± 0.9 b	7.94 ± 0.2 c	1.69 ± 0.2 a	1.54 ± 0.03 a	13.7 *± 0.5 b	2.72 ± 0.1 a	4.66 * ± 0.2 b	2.05 ± 0.3 a
Site II		16.4 ± 1.4 a	5.29 ± 0.1 b	9.89 ± 0.1 c	0.85 ± 0.03 a	0.01 ± 0.0 a	3.09 * ± 0.3 b	2.7 * ± 0.02 a	1.10 ± 0.3 b	1.55 ± 0.2 b
Site III		32.5 * ± 0.2 a	2.74 ± 0.4 b	64.7 ± 0.2 c	2.04 ± 0.1 a	3.02 ± 0.4 a	8.19 * ± 0.7 b	1.45 ± 0.3 a	1.37 ± 03 a	2.80 * ± 0.3 b
Site I	Cu	2.70 ± 0.4 a	4.04 ± 0.1 b	2.47 ± 0.04 a	4.53 ± 0.1 a	2.54 ± 0.03 b	7.63 * ± 0.03 c	5.82 * ± 0.1 a	1.69 ± 0.1 b	2.48 ± 0.2 c
Site II		10.9 ± 0.6 a	5.64 ± 0.1 b	3.81 ± 0.04 c	13.5 * ± 1.5 a	2.99 ± 0.1 b	6.23 ± 0.03 b	4.27 * ± 1.1 a	2.8 ± 0.01 ab	1.16 ± 0.1 b
Site III		23.3 ± 0.4 a	4.89 ± 0.1 b	23.4 ± 0.2 a	3.19 ± 0.0 a	2.33 ± 0.1 b	3.81 * ± 0.1 c	12.7 * ± 4.7 b	3.52 ± 0.2 a	3.56 ± 0.3 a
Site I	Mn	11.6 ± 0.5 a	228.5 * ± 0.9 b	109 ± 1.4 c	3.64 ± 0.03 a	12.5 * ± 0.9 b	6.48 ± 0.3 c	9.77 ± 2.8 a	28.7 * ± 4.1 b	20.1 ± 1.6 ab
Site II		216 ± 0.7 a	230.5 * ± 0.3 b	204.5 ± 1.1 c	4.52 ± 0.04 a	15.02 * ± 0.1 b	6.48 ± 0.3 c	9.62 ± 2.1 a	25.4 ± 0.6 ab	54.4 * ± 14.4 b
Site III		268 * ± 1.1 a	161.2 ± 0.4 b	201.9 ± 0.1 c	5.85 ± 0.14	6.44 * ± 0.3	5.87 ± 0.08	9.36 ± 1.5 a	30.7 * ± 3.9 b	25.0 ± 2.3 b
Site I	Zn	13.7 ± 1.9 a	33.4 ± 3.7 b	47.4 * ± 0.8 c	24.41 ± 0.3 a	13.95 ± 1.1 b	33.4 * ± 0.3 c	23.2 ± 1.6 ab	26.4 * ± 3.1 a	13.8 ± 3.0 b
Site II		65.6 ± 3.3 a	11.5 ± 2.0 b	5.78 ± 0.5 b	50.6 * ±11 a	15.44 ± 2.0 b	30.4 ± 0.3 ab	30.7 ± 0.4 a	40.2 * ± 1.0 a	17.6 ± 4.2 b
Site III		81.2 ± 0.7 a	10.5 ± 0.9 b	105.4 * ± 0.6 c	26.4 * ± 0.9 a	13.37 ± 0.9 b	19.1 ± 1.1 c	27.5 ± 0.9 a	23.0 ± 1.7 a	39.8 * ± 0.4 b
Site I	Fe	5516.3 ± 144 a	15,016 * ± 39 b	5178.6 ± 115 a	919 * ± 13 a	162.0 ± 4.4 b	115.9 ± 0.7 c	40.2 ± 3.9 a	217 * ± 16 b	168.4 ± 16.2 b
Site II		14,492.8 ± 692	14,793.5 * ± 321	13,697.7 ± 10.9	55.2 ± 13 a	113.3 ± 13.7 b	205.6 * ± 0.6 c	120.9 ± 21 a	152 * ± 2.2 a	147.7 ± 47.0 a
Site III		17,389.5 * ± 171 a	9354.7 ± 82 b	14,201.3 ± 257 c	228.9 ± 1.7 a	199.2 ± 2.6 b	243.4 * ± 9.8 a	148.8 ± 32 b	159.7 * ± 24 b	126.1 ± 3.0 a

**Table 2:** Na^+^ and K^+^ (g kg^−1^) content in soil, root, and leaves of *Salvadora persica* at Sandspit (Site I), Clifton (Site II) and Korangi (Site III). Different letters on error bars indicate significant differences among season on the same site (*n* = 3) at *p* ≤ 0.05. (Bonferroni/Games-Howell tests wherever applicable).

		Soil			Root			Leaves		
Sites	Metal	Winter	Summer	Post Monsoon	Winter	Summer	Post Monsoon	Winter	Summer	Post Monsoon
Site I	Na^+^	2.96 ± 0.1 a	2.82 ± 0.1 a	9.32 ± 0.2 b	44.7 ± 0.1 a	9.59 ± 0.45 b	111.6 ± 7.2 c	11.9 ± 4.7 a	24.4 ± 1.5 a	90.5 ± 3.5 b
Site II		1.19 ± 0.0 a	13.5 ± 0.5 b	8.16 ± 0.2 c	11.0 ± 0.9 a	11.5 ± 2.0 a	30.6 ± 0.7 b	10.6 ± 0.7	34.4 ± 4.3	53.6 ± 26.5
Site III		2.01 ± 0.0 a	44.4 ± 5.8 b	13.8 ± 1.1 a	11.0 ± 0.01 a	5.68 ± 0.5 b	8.61 ± 1.2 ab	40.6 ± 7.1	38.2 ± 2.1	40.7 ± 0.7
Site I	K^+^	0.28 ± 0.01 a	0.85 ± 0.0 b	0.65 ± 0.0 c	7.88 ± 0.2 ab	5.64 ± 0.5 a	17.8 ± 4.3 b	4.19 ± 0.3 a	11.3 ± 0.5 b	10.32 ± 1.1 b
Site II		2.15 ± 0.3	1.91 ± 0.5	2.75 ± 0.01	9.26 ± 0.3 a	6.57 ± 0.0 a	14.1 ± 1.1 b	7.23 ± 0.3 a	9.39 ± 0.6 b	8.33 ± 0.1 ab
Site III		2.66 ± 0.0 a	1.22 ± 0.1 a	6.52 ± 1.1 b	7.83 ± 0.1 a	2.82 ± 0.01 b	7.38 ± 0.6 a	9.26 ± 0.9	9.39 ± 0.6	10.34 ± 1.1

**Table 3:** Leaf Water relation parameters (-MPa) derived from PV-curves of *Salvadora persica* at Sandspit (Site I), Clifton (Site II) and Korangi (Site III). *ΨW*_o_ = Water potential at full turgor; *ΨS*_o_ = Osmotic potential at full turgor; *ΨTLP =* Water potential at turgor loss point and *ε = Bulk elastic modulus* and Leaf Succulence (mg g^−1^ DW). Different letters on error bars indicate significant differences among seasons on the same site (*n* = 3) at *p* ≤ 0.05 (Bonferroni/Games-Howell tests wherever applicable). Asterisk (*) in mean values represent the most significant data.

Sites	Parameter	Winter	Summer	Post monsoon
Site I	*ΨW*_o_	1.2 ± 0.1 a	1.65 * ± 0.2 b	1.1 ± 0.1 a
Site II		1.8 * ± 0.2 b	1.4 ± 0.2 a	1.6 ± 0.2 b
Site III		1.5 ± 0.3 a	2.0 * ± 0.04 b	1.8 ± 0.1 ab
Site I	*ΨS*_o_	1.8 ± 0.3 a	2.3 * ± 0.2 b	1.75 ± 0.2 c
Site II		2.3 ± 0.2 b	1.9 ± 0.1 a	2.4 * ± 0.3 b
Site III		2.5 * ± 0.2 b	1.8 ± 0.4 a	2.4 * ± 0.2 b
Site I	*ΨTLP*	2.7 ± 0.4 a	3.2 * ± 0.1 b	2.8 ± 0.04 a
Site II		3.6 * ± 0.4 b	2.8 ± 0.2 a	3.4 ± 0.2 b
Site III		3.8 * ± 0.3 b	2.8 ± 0.1 a	3.6 ± 0.2 b
Site I	*ε*	6.4 ± 0.5 a	7.0 ± 0.9 b	6.6 ± 1.4 c
Site II		6.2 ± 0.2 b	5.0 ± 0.3 a	4.8 ± 0.3 a
Site III		7.6 * ± 1.1 c	6.1 ± 0.4 b	5.5 ± 0.3 a
Site I	*SUC*	3.1 ± 0.5 b	2.9 ± 0.2 a	3.0 ± 0.4 b
Site II		4.2 ± 0.2 a	5.5 * ± 0.3 b	4.9 ± 0.3 ab
Site III		3.6 ± 0.2 a	4.7 * ± 0.3 c	4.1 ± 0.3 a

## Data Availability

All data is available in the work/Supplementary Material referenced.

## Supplementary Materials

The following supporting information can be downloaded at: https://www.mdpi.com/article/10.3390/plants12030598/s1, Figure S1: Translocation factor (TF) and Bioconcentration factor (BCF) of Salvadora persica at the study sites Sandspit (Site I), Clifton (Site II) and Korangi (Site III).
