# Supplementary material for: Phytoremediation of Potentially Toxic Elements from Contaminated Saline Soils Using Salvadora persica L.: Seasonal Evaluation
Source: Plants (Basel). 2023 Jan 29;12(3):598. doi: 10.3390/plants12030598 (PMC9920363; doi:10.3390/plants12030598)

Supplementary Figure S1: Translocation factor (TF) and Bioconcentration factor (BCF) of Salvadora persica at the study sites Sandspit (Site I), Clifton (Site II) and Korangi (Site III).

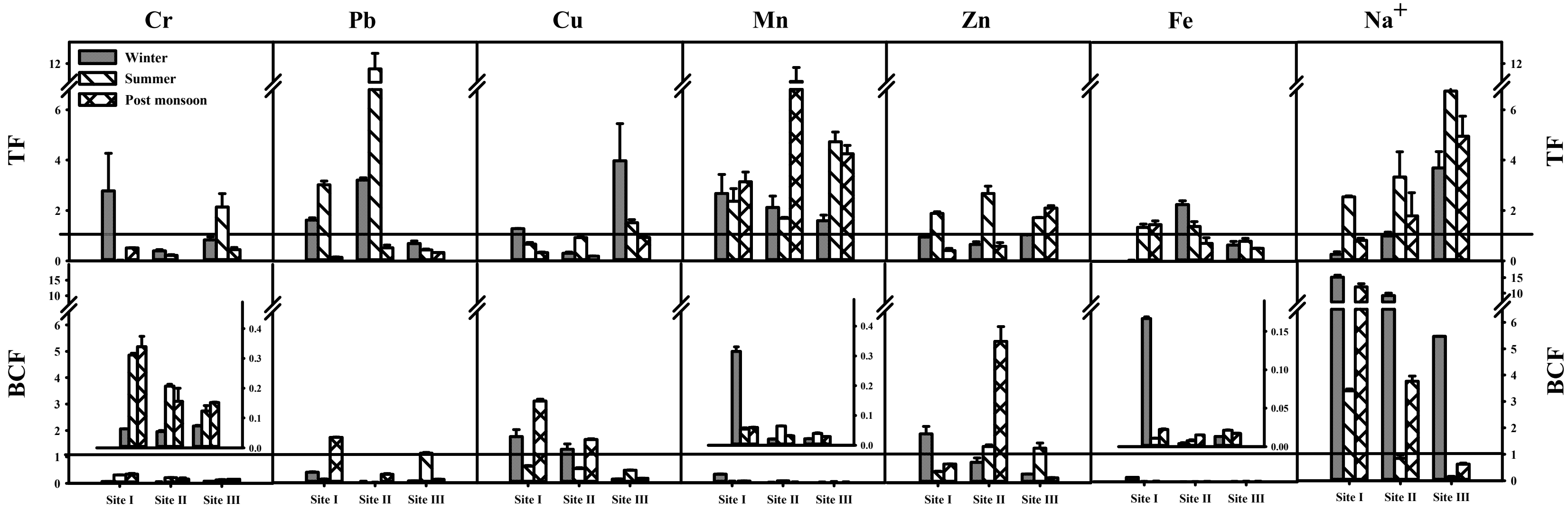

Supplement: Supplementary file 1 [file plants-12-00598-s001.zip › plants-2164396-supplementary.pdf]
